# *Allium porrum* Extract Decreases Effector Cell Degranulation and Modulates Airway Epithelial Cell Function

**DOI:** 10.3390/nu11061303

**Published:** 2019-06-08

**Authors:** Sara Benedé, Ana Gradillas, Mayte Villalba, Eva Batanero

**Affiliations:** 1 Departamento de Bioquímica y Biología Molecular, Facultad de Ciencias Químicas, Universidad Complutense de Madrid, 28040 Madrid, Spain; mvillalba@quim.ucm.es; 2 Cembio (Centro de Metabolómica y Bioanálisis), Departamento de Química y Bioquímica, Facultad de Farmacia, Universidad CEU San Pablo, 28668 Monteprincipe, Spain; gradini@ceu.es

**Keywords:** *Allium porrum*, antioxidant activity, phenolic compounds, mast cell, epithelium

## Abstract

*Allium* genus plants, such as leek (*Allium porrum*), are rich sources of anti-inflammatory and anti-oxidant secondary metabolites; this is of interest because it demonstrates their suitability as pharmacological alternatives for inflammatory processes, including allergy treatment. The composition of methanolic leek extract (LE) was analyzed by GC–MS and LC–IT/MS, and the total phenolic content and antioxidant capacity were quantified by colorimetric methods. Its pharmacological potential was analyzed in human bronchial epithelial Calu-3 cells, human mast cells LAD2, and humanized rat basophiles RBL-2H3. LE exhibited a cytotoxic effect on Calu-3 cells and HumRBL-2H3 cells only at high concentrations and in a dose-dependent manner. Moreover, LE decreased the degranulation of LAD2 and HumRBL-2H3 cells. LE treatment also significantly prevented alterations in transepithelial electrical resistance values and mRNA levels of glutathione-S-transferase (GST), c-Jun, and NFκB after treatment with H_2_O_2_ in ALI-cultured Calu-3 cells. Finally, ALI-cultured Calu-3 cells treated with LE showed lower permeability to Ole e 1 compared to untreated cells. A reduction in IL-6 secretion in ALI-cultured Calu-3 cells treated with LE was also observed. In summary, the results obtained in this work suggest that *A. porrum* extract may have potential anti-allergic effects due to its antioxidant and anti-inflammatory properties. This study provides several important insights into how LE can protect against allergy.

## 1. Introduction

Plants have been used for medicinal purposes since prehistoric times. They synthetize a wide variety of active compounds, named phytochemicals, with a potential use in drug development. Over the last few years, there has been an increase in the demand and recognition of medicinal plants. In fact, since 1999, the World Health Organization (WHO) has published three monographs to provide scientific information on the safety, efficacy, and quality control of widely used medicinal plants [1].

To date, the World Allergy Organization (WAO) has estimated that between 10–40% of the global population is affected by some type of allergic diseases, and it is predicted that their prevalence will continue to increase worldwide, thus representing a major global public health issue [2]. These inflammatory diseases are characterized by an exacerbated T helper 2 (Th2)-type immune response and the production of high levels of serum immunoglobulin E (IgE) against substances (named allergens) usually harmless to most people. Mast cells are the main effector cells responsible for allergic symptoms as they release multiple pro-inflammatory mediators. Moreover, growing evidence has demonstrated the key role of epithelial cells in orchestrating and influencing allergic responses, and the association between epithelial barrier dysfunction and allergies. Thus, mast cells represent promising therapeutic targets for the treatment of allergic diseases. Corticosteroids are usually administered to control these pathological processes, however, long-term steroid treatment could cause side effects such as hypertension, cataracts, and osteoporosis [3]. Drugs such as ketotifen and sodium cromoglycate (a khellin derivative obtained from the *Ammi visnaga* plant [4]) are the most commonly used mast cell stabilizers for the treatment of bronchial asthma and allergic conjunctivitis [5].

The relative efficacy of these drugs, together with their side effects and high cost, raise the urgent need to look for new bioactive compounds. Searching for new stabilizers of mast cells that prevent their activation and the subsequent releasing of pro-inflammatory mediators contained in their granules is one of the main goals of allergy treatment. Recent advances towards the discovery of the next generation of mast cell stabilizers include a phytochemical study of plant extracts such as phenolic compound [4], while promising results have been obtained for magnolol and honokiol [6], resveratrol [7], and curcumin [8]. Furthermore, Kimata et al. showed that flavonoid luteolin inhibits the secretion of the pro-inflammatory cytokines TNF-α and IL-6 in mouse mast cells [9]. Among the phenolic compounds, it has been shown that ellagic acid from fruits and nuts and curcumin from turmeric attenuate the IgE-mediated allergic response in vitro and in vivo [8]. The L-amino acid theanine of green tea inhibits the secretion of pro-inflammatory cytokines in human mast cells by suppressing the activation of the nuclear transcription factor NFκB [10]. Finally, terpenoids [11], alkaloids [12], and phenols [6] from different plants, also exhibit properties suitable for allergy treatment.

Moreover, oxidative stress induced from environmental factors can contribute to allergic inflammatory response. In that case, antioxidant phytochemicals such as the flavonoids morin flavonoid—present in many herbs—or astragalin from green tea seeds and persimmon leaves may reduce airway inflammation by regulating reactive oxygen species (ROS) signaling [13]. Previous studies have reported that phytochemicals from *Allium* genus plants—in particular *A. cepa* (onion) and *A. sativum* (garlic)—exhibit beneficial properties for human health; anti-microbial, antioxidant, anti-tumorigenic, and immunomodulatory properties have been found [14,15,16,17,18]. Regarding allergic diseases, garlic extract protects mice against allergic inflammation of the airways [19].

Leek (*A. porrum*) is a hardy biennial crop that has been historically consumed since ancient Egypt, and today, it is widely used as a culinary ingredient in gastronomy throughout the world. A broad set of bioactive plant chemicals has been reported in leek [20,21]; thus, it is a promising plant in the search for new phytochemicals to treat allergy.

In this study, we investigated the effect of leek extract (LE) on both human bronchial epithelial and mast cells. Individual phytochemicals were identified in the methanolic LE and their antioxidant, anti-inflammatory, membrane permeability, and cytotoxic activities were assayed.

## 2. Materials and Methods

### 2.1. Chemicals 

A collection of a set of 48 phenolic compounds meeting commercially available standards was purchased from Sigma Aldrich Chemicals. All of them were of an analytical grade and were used without further purifications. Methanol (mass spectrometry grade) was purchased from Fluka and ultrapure water was obtained from Milli-Q apparatus. The standard solutions (10 µg/mL) were prepared in methanol.

### 2.2. Plant Material

Leeks (*Allium ampeloprasum* var. *porrum*) were collected from the fields of Jaca (Spain) in autumn 2016. Long white stems (approximately 200 g) were skinned, cut longitudinally into thin strips, freeze-dried, and then ground into powder. Samples were stored in sealed plastic bottles at −80 °C until used. The absence of LPS in the samples was confirmed by using the transfected cell line THP1-XBlue™ and the QUANTI-Blue™ assay (InvitroGen), following manufacturer instructions.

### 2.3. Extract Preparation by Ultrasonication

Leek extract (LE) was prepared by ultrasonication of dried samples (10% *w*/*v*) in 100% methanol using a Brason Ultrasonic Bath Model 1200 (Branson, MO, USA) at room temperature for 30 min. After centrifugation at 5000× *g* for 15 min, samples were filtrated through a 0.4 µm filter, followed by a 0.2 µm filter. Three milliliters of liquid extract were used for yield determination. Solvent was removed in a Univapo 100H (Biotech S.L., Spain) vacuum centrifuge at room temperature. Dry extracts were stored at −80 °C to prevent oxidative damage until analysis.

### 2.4. Determination of Total Phenols Content

Total phenols were determined using Folin–Ciocalteu’s method by Radovanović et al. with minor modifications, using gallic acid as the standard [22]. Briefly, LE (100 µL) was mixed with 300 µL of Folin–Ciocalteu’s reagent and 600 µL of NaHCO_3_. After 1 h at room temperature, the absorbance was measured at 765 nm using a UV-VIS 1800 spectrophotometer (Shimadzu, Japan). Total phenols were expressed as mg of gallic acid equivalents (GAE) per g dry weight and presented as the mean ± standard error of mean (SEM) of triplicate samples.

### 2.5. Antioxidant Activity

#### 2.5.1. Determination of Total Antioxidant Activity

The total antioxidant activity of the LE was determined by the phosphomolybdenum method [23] based on the reduction of Mo (VI) to Mo (V) by the antioxidants present in the sample. An aliquot of 100 μL of LE was incubated with 1 mL of reagent solution (H_2_SO_4_ 0.2 M, NaH_2_PO_4_ 9.3 mM, (NH_4_)_6_MO_7_O_24_ 1.3 mM) for 90 min at 95 °C. After cooling to room temperature, the increase of absorbance by the subsequent formation of a green phosphate/Mo (V) complex was measured at 695 nm in a UV-VIS 1800 spectrophotometer. Total antioxidant capacity was expressed as mg of ascorbic acid equivalents (AAE) per g of dry weight. Data were the mean values ± SEM of triplicate experiments.

#### 2.5.2. Determination of DPPH Free Radical Scavenging Activity

The radical scavenging activity (RSA) of the LE was determined by using the 2,2-Diphenyl-2-picrilhydrazil (DPPH) assay according to Brand-Williams et al., with some modifications [24]. Briefly, 100 μL aliquots of LE (0.01–1 mg/mL) were incubated with 100 μL of DPPH (80 μg/mL) in methanol for 30 min in the dark at room temperature. The decrease in the absorbance of the DPPH solution after the addition of an antioxidant was measured at 517 nm in an iMark plate reader (Biorad, Hercules, CA, USA). Ascorbic acid was used as reference. The percentage (%) of RSA was calculated according to the following equation:

%RSA = [(Abs_Control_ − Abs_Sample_)/Abs_Control_] × 100 (1)

where Abs_Control_ and Abs_Sample_ are the absorbances of the DPPH radical in the absence and presence of LE, respectively. The effective concentration of the extract required to neutralize 50% (w/v) of DPPH free radicals (EC50) was expressed in μg/mL and interpolated from the percent inhibition plot against the extract concentration. Data were the mean values ± SEM of triplicate experiments.

### 2.6. Gas Chromatography–Mass Spectrometry (GC–MS) Analysis

The analysis of the LE by GC–MS was carried out at the mass spectrometry research support center at the Complutense University of Madrid (Spain) following a standard method. Briefly, samples were dried under nitrogen and derivatized with 60 μL of N,O-Bis(trimethylsilyl)trifluoroacetamide and trimethylchlorosilane (BSTFA: TMSC; 99:1). Then, the derivatized samples were analyzed on a Varian CP3800 GC system (Varian Inc., United States) with a ZB-5MS plus column (Phenomenex Inc., 30 m × 0.25 mm × 0.25 μm) coupled to a Saturn 2200 ion trap mass spectrometer (Varian Inc., Palo Alto, CA, USA), using helium as the starting mobile phase at 60 °C, gradually increasing to 220 °C at 16 min, and to 320 °C at 5 min, with 320 °C held for 6 min. The injection volume was 2 μL and the flow rate was set up to 1 mL/min. Compounds were identified by searching against the NIST library and considering an R-Match higher than 700.

### 2.7. Liquid Chromatography–Mass Spectrometry (LC–IT/MS)

Three biological samples of dry extract from *A. porrum* (obtained as previously described in Section 2.3) were prepared as follows: 300 µL of methanol were added to 30 mg of powder. The mixture was vortexed for 2 min, sonicated for 10 min at room temperature in an ultrasonic bath and finally, centrifuged at 10,000× *g* for 5 min at 4 °C. Supernatants of two replicates of each biological sample were then collected for direct analysis.

Phenolic acids and flavonoids were then analyzed by LC–IT/MS. The identification of compounds was performed using an Agilent 1100 HPLC system (Agilent Technologies, Germany) connected to a Bruker Daltonics esquire 3000^plus^ Ion Trap (IT) Mass Spectrometer (Bruker Daltonics, Germany) with an Electrospray Interfase (ESI).

With regard to the LC analysis, separation was on a Zorbax Eclipse XDB-C18 column (4.6 × 50 mm, 1.8 µm, Agilent Technologies) running the following gradient of methanol (solvent B) versus 0.1% (v/v) aqueous formic acid (solvent A) as the starting mobile phase: 2% B (0–1 min), 2–50% B (1–13 min), 50–95% B (13–18 min), 95% B for 2 min (18–20 min), and returning to starting conditions 2% B in 1 min (20–21 min) to finally keep the re-equilibration with a total analysis time of 25 min [25]. The injection volume was 10 μL, the flow rate was 0.5 mL/min, and the column temperature was 60 °C. Peaks were detected by UV/visible light absorbance collecting chromatograms at 260 nm. 

With regard to the MS analysis, the ESI–IT/MS was conducted in a positive and negative ionization mode and operated according to defined conditions: Nitrogen gas temperature was 350 °C; drying gas flow rate was 11.5 mL/min; capillary voltage was ± 4000 V and nebulizing pressure was 25 psi. Mass spectra were recorded using the full scan mode in the range of *m*/*z* 50–1300. 

For the study, the data acquisition software employed was Esquire Control 5.2 and the data analysis was performed using the DataAnalysis 3.1 software (Bruker Daltonics).

### 2.8. Cell Culture

A human bronchial epithelial Calu-3 cell line (2.5 × 10^5^ cells/mL, ATCC No. HTB-55, Lot. 61449062) was grown in DMEM/F-12 (Dulbecco’s Modified Eagle Medium/F-12 Nutrient Mixture, Gibco) supplemented with 10% (*v*/*v*) fetal bovine serum, 100 IU/mL penicillin, 100 μg/mL streptomycin, and 2 mM L-glutamine, at 37 °C and 5% CO_2_ until confluence. Then, the cells (7.5 × 10^5^ cells/mL) were grown at an air-liquid interface (ALI) on inserts (24-well plates with a pore size of 0.4 μm and a surface of 0.33 cm^2^, Corning) in DMEM F-12 supplemented with 5% (*v*/*v*) fetal bovine serum, 100 IU/mL of penicillin, 100 μg/mL of streptomycin, and 2 mM of L-glutamine. Culturing cells at ALI led to the formation of polarized tight monolayers, which mimic a human respiratory epithelial barrier.

ALI-cultured Calu-3 cells were apically exposed to the LPS free-Ole e 1 (25 µg/mL) allergen or LE (5 μg/mL) for 16 h, or H_2_O_2_ 1mM for 24 h, in a DMEM F-12 supplemented medium on days 2 or 7, which correspond to a non-differentiated and differentiated epithelium, respectively. Ole e 1 was purified from olive pollen (*Olea europaea*, Iberpolen, Spain) as previously described [26]. Epithelial barrier integrity was checked by measuring the transepithelial electrical resistance (TEER) using an EVOM2 device (WPI).

The human mast LAD2 cell line was kindly provided by Drs. Dean Metcalfe and Arnold Kirshenbaum (National Institute of Allergy and Infectious Diseases, NIH, Bethesda, MD, USA) [27]. Cells (0.25–5 × 10^5^ cells/mL) were grown in serum-free Stem Pro-34 media (Invitrogen, Carlsbad, CA, USA), containing nutrient supplements, 2 mM L-glutamine, 100 IU/mL penicillin, 100 µg/mL streptomycin, and 100 ng/mL recombinant human stem cell factor (Peprotech, London, UK) at 37 °C and 5% CO_2_. The culture medium was hemidepleted each week with a fresh medium.

Rat basophilic leukemia cells transfected with cDNA coding for the human high affinity IgE receptor chains (HumRBL-2H3) were kindly donated by Dr. Lothan Vogel (Division of Allergology, Paul-Ehrlich-Institut, Langen, Germany). The cells were cultured in MEM supplemented with 5% fetal bovine serum, 100 UI/mL penicillin, and 100 µg/mL streptomycin. The cells (5 × 10^4^ cells/mL) were grown in 75 cm^2^ tissue culture flasks at 37 °C and 5% CO_2_ as previously described [28].

### 2.9. Cytotoxicity Assay

Dry LE was dissolved at 10 mg/mL in a phosphate buffer with pH 7.4, and the possible cytotoxic effects on LAD2, HumRBL-2H3, and Calu-3 cell lines were investigated by conducting cell viability assays using two-fold serial dilutions. Cell viability was determined by the 3-(4,5-dimethylthiazol-2-yl)-2,5-diphenyltetrazolium bromide (MTT) assay [29]. Cells were seeded in 96-well microtiter plates for 24 h at 37 °C and 5% CO_2_ at the indicated density: HumRBL-2H3 cells, 5 × 10^3^ cells per well; LAD2 cells, 4.5 × 10^4^ cells per well; and Calu-3 cells, 3.0 × 10^4^ cells per well. Then, various concentrations of LE were added to the cells and incubated for an additional 72 h. Twenty μl of MTT solution (5 mg/mL PBS) were added to each well. Samples were incubated for a further 4 h in the dark. Then, media were removed, and 100 μL of dimethyl sulfoxide:methanol (1:1) were added to dissolve the purple insoluble MTT formazan; the absorbance was measured at 570 nm in an iMark plate reader (Biorad, Hercules, CA, USA) using a 650 nm filter as a reference. Cell viability was expressed as the percentage (%) of dead cells relative to the untreated control cells. All determinations were performed in triplicate.

### 2.10. Interleukin (IL) 6 Analysis

The determination of human IL-6 levels in the supernatants of the ALI-cultured Calu-3 cells after exposure to Ole e 1 with or without LE was performed using the ELISA kit BD OptEIA (BD Biosciences, San Jose, CA, USA), according to the manufacturer instructions.

### 2.11. Epithelial Permeability Analysis

The permeability of ALI-cultured Calu-3 cells to the Ole e 1 allergen, in the presence of absence of LE, was analyzed by a Western blot using an anti-Ole e 1 rabbit polyclonal antiserum (1:5000) generated by Dr. F. Vivanco’s laboratory (Fundación Jiménez Díaz, Madrid, Spain), as previously described [30]. For that purpose, cells were apically exposed to Ole e 1 (25 µg/mL), with or without LE (5 µg/mL), for 7 h on day 7 at ALI. Basolateral and apical supernatants were loaded onto SDS-PAGE (15% acrylamide gel) and then electro transferred onto a nitrocellulose membrane using a Trans-Blot SD Semi-Dry Transfer Cell (Biorad, Hercules, CA, USA). Detection was achieved by means of enhanced chemiluminescence with Pierce ECL Western Blotting Substrates (Thermo Scientific, Waltham, MA, USA) according to the manufacturer’s instructions. Images were acquired in a UVP ChemiDoc-It (Fisher Scientific, Hampton, NH, USA). Ole e 1 was purified from olive pollen as described by Villalba et al. [26].

### 2.12. Degranulation Assay

Sera were collected from Ole e 1-allergic patients in accordance with a protocol approved by the ethics committee of both Ciudad Real and Cordoba Hospital (Spain), and a written informed consent was obtained from all subjects. The inclusion criteria were a well-defined clinical history of allergy to olive pollen and specific IgE antibodies to the Ole e 1 allergen. Non-atopic individuals were included as negative controls.

The assay was performed as previously described, with minor modifications [31]. HumRBL-2H3 cells (0.45 × 10^6^ cells/mL) were incubated in 96 well plates for 24 h at 37 °C under 5% CO_2_. Passive sensitization was performed by overnight incubation with sera from Ole e 1-allergic patients and non-atopic individuals as control (optimal dilution). After washing them by centrifugation at 200× *g* for 10 min, cells were treated with LE (5 µg/mL) or PBS for another 5 h. Degranulation of cells was induced by incubation with Ole e 1 (25 µg/mL) in Tyrode´s buffer with 50% of D_2_O for 30 min. The released β-hexosaminidase enzyme was measured in the supernatants using *p*-nitrophenyl-*N*-acetyl-β-D-glucosaminide (3.5 mg/mL, Sigma-Aldrich, Germany) in 40 mM citric acid, pH 4.5. Optical density was measured in an ELISA reader at 405 nm. Data were expressed as the percentage (%) of total β-hexosaminidase content in the cells. The spontaneous release was subtracted from this total value. 

For non-IgE mediated LAD2 activation, cells (0.2 × 10^6^) were seeded in 96 well plates and treated with LE (0.05–50 µg/mL) for 24 h. Then, cells were activated with compound 48/80 in Tyrode´s buffer at 5 μg/mL for 30 min and processed as described above.

### 2.13. RNA Extraction and Quantitative RT-PCR (qRT-PCR) Analysis

RNA was extracted from ALI-cultured Calu-3 cells using a Qiagen-RNeasy kit (Qiagen, CA, USA), according to the manufacturer’s instructions. RNA concentration and integrity were assessed in a 2100B Bioanalyzer (Agilent Technology). First-stranded cDNA was synthetized using the Superscript III first-strand synthesis System (Invitrogen, Carlsbad, CA, USA). RT–PCR was performed on a 7900HT fast real-time PCR detection system (Applied Biosystems), with the Power SYBR Green PCR Master Mix (Applied Biosystems) and the specific primers described in Table 1. Glyceraldehyde-3-phosphate dehydrogenase (GAPDH) was used as the housekeeping gene. Data were expressed asfold increase compared with levels measured in untreated cells by using a ∆∆CT threshold cycle method of calculation. All amplifications were carried out in triplicates.

### 2.14. Statistical Analysis

Statistical analyses were performed using GraphPad Prism software, version 7.0e (GraphPad). A two-tailed Student’s *t* test, 1-way ANOVA, or 2-way ANOVA were used for determining statistical significance (*p* < 0.05). Data were expressed as the mean ± SEM of three independent experiments. 

## 3. Results

### 3.1. Characterization of Leek Extract

Leek extract was obtained using methanol extraction with an average yield of 45.5% ± 1.5 from three independent extracts, and qualitative composition was studied by GC–MS. Twelve organic acids, several carbohydrates, and L-amino acids were identified (Table 2).

The total phenol content in LE was 2.3 ± 0.2 mg/g dry weight, expressed as GAE (Table 3).

The determination of phenolic compounds of LE was carried out by LC–IT/MS (Table 4). Performing a detailed evaluation of the mass spectra of each peak, and based on the fragments observed in the source, we identified several flavonoids glycosides and steroidal saponins. The metabolites found and putatively identified in the samples are summarized in the Table 3. They corresponded to the *m*/*z* experimental values from the base peak chromatograms obtained in the positive ion mode as well as the fragments generated in the source.

Besides, the fragmentation patterns observed for some compounds were consistent with the loss of hexose units and the corresponding aglycons. Based on the MS fragmentation data in the ESI positive mode, kampherol di- and triglycosides were predicted to produce kaempherol aglycone (*m*/*z* 287) after the loss of glycosyl units, while the quercetin mono-, di-, and triglycosides were predicted to produce quercetin aglycone (*m*/*z* 303) after the loss of glycosyl units.

The flavonoids (-)-epicatechin, (+)-catechin, kaempferol-*O*-diglycoside and *O*-triglycoside derivatives, and quercetin-*O*-triglycoside, quercetin 3′,4′-*O*-diglycoside, and quercetin-O-monoglycoside derivatives, were also detected in the LE.

### 3.2. Antioxidant Activity of Leek Extract

Using the phosphomolybdenum method, the total antioxidant activity of methanolic LE, expressed as mg of AAE per gram of dry extract, was 60.2 ± 3.1 (Table 3). Another indicator of the antioxidant activity of the LE was its free high radical scavenging activity of 289.6 ± 6.1 µg/mL (expressed in IC_50_ values) determined using DPPH. 

### 3.3. Leek Extract Exhibits Cytotoxic Activity in a Dose-Dependent Manner

**Figure 1:**
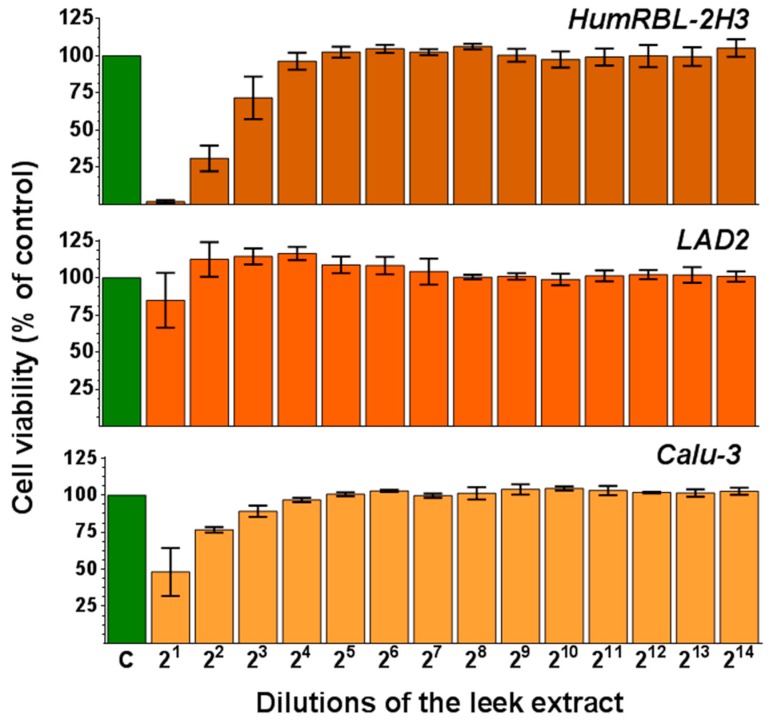
Cytotoxicity of methanolic LE at different concentrations on three cell lines: Humanized rat basophils (HumRBL-2H3), human mast cells (LAD2), and bronchial epithelial cells (Calu-3). The cell viability was assayed using the 3-(4,5-dimethylthiazol-2-yl)-2,5-diphenyltetrazolium bromide (MTT) method after 72 h of treatment. DMSO was used as positive control. Data are expressed as a percentage (%) of control (C) and shown as the mean ± SEM of three independent experiments.

In order to evaluate the cytotoxic effect of the methanolic LE, a cell viability assay was performed on three cell lines using MTT: Humanized rat basophils (HumRBL-2H3), human mast LAD2 cells, and human bronchial epithelial cells (Calu-3). The results are shown in Figure 1. No cytotoxic effect was observed for the LE on any of the tested cell lines at dilution ≥2^4^. However, the cytotoxicity of the LE increased as the concentration did. At 2^2^ dilution, LE showed the most cytotoxic activity on HumRBL-2H3 cells (cell viability was reduced to 25%), followed by Calu-3 (75% cell viability). No significant cytotoxic effect of LE was detected on the LAD2 cells at any of the tested concentrations.

### 3.4. Leek Extract Decreases Degranulation of Mast Cells

**Figure 2:**
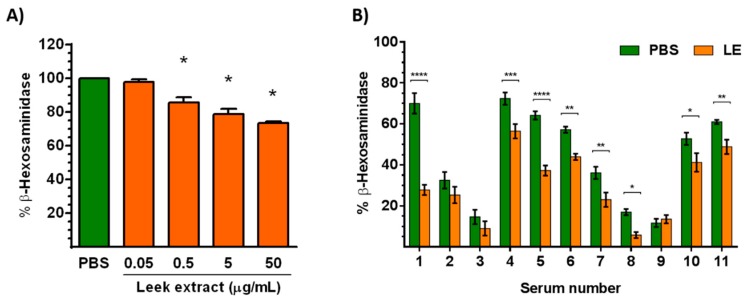
Measurement of β-hexosaminidase after the activation of (**A**) human mast cells LAD2 with the compound 48/80 (5 μg/mL) and (**B**) HumRBL-2H3 with Ole e 1 (25 ng/mL) after treatment with leek extract (LE) −0.05–50 μg/mL (**A**) or 5 μg/mL (**B**) for 48 h. HumRBL-2H3 were previously sensitized with sera from patients allergic to Ole e 1. * *p* < 0.05, ** *p* < 0.01, *** *p* < 0.001, **** *p* < 0.0001 compared to control cells treated with PBS.

To assay the effect of LE on degranulation of effector cells, the human mast cell line LAD2 was treated with LE for 48 h before addition of the component 48/80, a polymer that promotes the activation and degranulation of mast cells through an IgE-independent pathway. As shown in Figure 2A, LE decreased the degranulation of LAD2 cells, expressed as the percentage (%) of β-hexosaminidase release, in a dose-dependent manner.

Leek extract also decreased the IgE-mediated degranulation of HumRBL-2H3 cells promoted by the Ole e 1 allergen in eight out of eleven individual sera tested from olive pollen-allergic patients (Figure 2B). 

### 3.5. Leek Extract Prevents Both the Decrease of TEER and Gene Expression Induced by H_2_O_2_ Oxidative Stress/Inflammatory Stimulus

**Figure 3:**
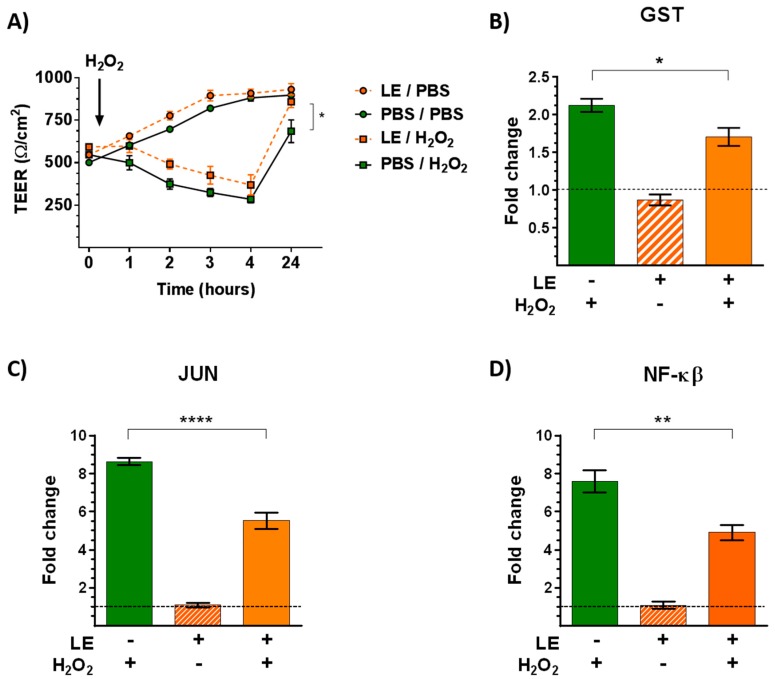
(**A**) Effect of leek extract on the response of epithelial cells to oxidative stress. On day 7, ALI-cultured Calu-3 cells were grown in the presence or absence of leek extract (LE, 5 μg/mL) for 48 h and then exposed to 1 mM H_2_O_2_. The time course of transepithelial electrical resistance (TEER) was measured. PBS, control cells. * *p* < 0.05 mRNA levels of GST (**B**), c-Jun (**C**) and NFκB (**D**) in ALI-cultured Calu-3 cells exposed to 1mM H_2_O_2_ for 48 h, after treatment with leek extract (5 μg/mL, LE) compared to control cells (PBS). Relative mRNA levels were determined by RT–PCR and expressed as fold increase of control values after normalization using GADPH as the housekeeping gene. Data are mean ± SEM of three different experiments. * *p* < 0.05, ** *p* < 0.01, **** *p* < 0.0001.

Pulmonary epithelium constitutes a protective physical and immunological barrier against exogenous substances. The effect of LE on epithelial barrier integrity was analyzed in ALI-culturedCalu-3 cells after exposure to H_2_O_2_ oxidative stress/inflammatory stimulus on day 7. Changes in TEER values (an indirect measure of intercellular apical junction formation) were monitored at different time-points post-treatment (Figure 3A). TEER values were not significantly higher on cells treated with LE compared to control cells. Treatment of cells with 1 mM H_2_O_2_ induced a decrease to 70% of control TEER values in a time-dependent manner. LE pretreatment protected against the deleterious effect of H_2_O_2_ on TEER values and seemed to promote epithelial barrier maturation.

To know more about the mechanism of action of LE, the expression of genes implicated in redox metabolism was analyzed by RT–PCR (Figure 3B–D). Leek extract treatment did not modify the expression levels of glutathione S-transferase (GST), c-Jun, and nuclear factor kappa B (NFĸB) mRNA in comparison to control cells. As expected, exposure to H_2_O_2_ increased mRNA levels of the three studied genes. However, this effect on mRNA levels was significantly prevented by LE, suggesting that LE can regulate the bronchial epithelial response induced by oxidative stress/inflammatory stimuli.

### 3.6. Leek Extract Decreases Epithelial Permeability to an Allergen

**Figure 4:**
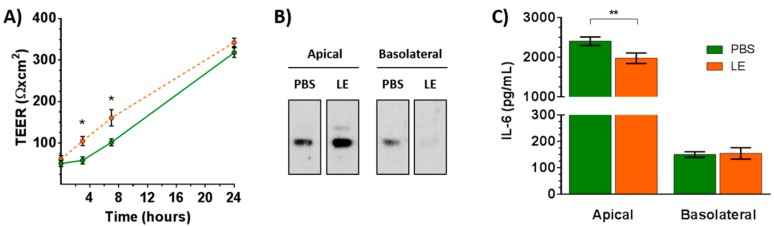
Effect of leek extract on the bronchial permeability to Ole e 1. (**A**) Time course of TEER values on day 2 ALI-cultured Calu-3 cells after treatment with leek extract (LE) compared to control cells (PBS). Data are the mean ± SD of triplicate determinations. * *p* < 0.05. (**B**) ALI-cultured Calu-3 cells were exposed to Ole e 1 (2.5 µg) on day 2 for 7 h after treatment with leek extract (LE, 5 μg/mL) for 24 h, and the presence of the allergen was determined in the medium using a rabbit polyclonal antibody. (**C**) ALI-cultured Calu-3 cells were treated with leek extract at 5 μg/mL (LE) for 24 h on day 2, and IL-6 levels in the medium were determined by sandwich ELISA. PBS, control cells. Data are the mean ± SD of triplicate determinations ** *p* < 0.01.

Epithelial permeability to Ole e 1, the main allergen of olive pollen, was analyzed to determine whether the positive effect of LE on the physical barrier was associated with a decrease in its permeability to the allergen. For this purpose, ALI-cultured Calu-3 cells were apically exposed to Ole e 1, in the presence or absence of LE, on day 2, which mimicked an impaired barrier as indicated by the low TEER values (Figure 4A). After 7 h, the presence of the allergen in the apical and basolateral media was detected by a Western blot using a specific anti-Ole e 1 polyclonal antiserum (Figure 4B). ALI-cultured Calu 3-cells displayed a disrupted physical barrier that exhibited permeability to Ole e 1. Interestingly, pretreatment with LE reduced permeability of Calu-3 cells to the allergen: No allergen was detected on the basolateral medium of treated cells. These results correlated with the significantly higher TEER values exhibited by cells treated with LE. Taken together, these data supported that LE has a protective effect on the bronchial physical barrier and thus may be useful in preventing the sensitization to allergens and the exacerbation of allergic diseases.

### 3.7. Leek Extract Decreases the Apical Release of IL-6 by Bronchial Epithelial Cells

We analyzed the effect of LE on the IL-6 level released by ALI-cultured Calu-3 cells into both apical and basolateral media after 24 h on day 2. The apical IL-6 level was significantly lower in cells treated with LE compared to control cells (Figure 4C). No differences were detected in basolateral media.

## 4. Discussion 

Species from the *Allium* genus- including *A. cepa* (onion), *A. sativum* (garlic), *A. ascalonicum* (shallot), and *A. porrum* (leek) are rich sources of secondary metabolites [33,34]. Among them, phenolic compounds are one of the most abundant metabolites, which exhibit anti-inflammatory and antioxidant properties [35,36]. Several studies have suggested that this type of compounds has a therapeutic potential to treat different diseases, including allergy [37].

Leek extract was obtained using methanol extraction since it has been reported as one of the best solvents to obtain plant materials in terms of yield, total phenol content, and antioxidant activity [38]. The qualitative composition of LE included twelve organic acids such as boric acid, fumaric acid, stearic acid, and oleic acid, for which antioxidant activity has been previously reported [39,40,41,42]. Several types of sugar—such as D-glucose and D-fructose—have also been identified; these represent a fast energy source. The L-amino acids valine and proline were identified, and their immunostimulatory properties have been shown in previous studies [43,44]. In addition, it has been reported that many compounds from leek protect against various diseases through their antioxidant activity, being able to chelate metals or to neutralize electron-stealing reactions involving free radicals [45].

The total phenol content found in LE (2.3 ± 0.2 mg/g dry weight) was lower than previously reported by other authors. This difference can be explained in terms of genetic, environmental, and experimental factors such as plant variety, geographical region, climatic conditions or extraction procedure, among others. Bernaert et al. found that total phenol content varied from 5 to 15 mg GAE/g dry weight in the extract of the 30 leek varieties analyzed [36]. Moreover, Piluzza and Bullitta reported that the total phenol content of onion and garlic extracts—two species belonging to the *Allium* genus—were 3.80 ± 0.42 mg EAG/g dry weight and 3.18 ± 0, 16 mg EAG/g dry weight, respectively [46].

Several flavonoids glycosides and steroidal saponins were found in LE. Beneficial effects on human health have been reported for phenolic components attributed to their antioxidant activity due to their redox properties, which can play an important role in absorbing and neutralizing free radicals, quenching singlet and triplet oxygen, or decomposing peroxides [47,48,49].

We found a high antioxidant activity of methanolic LE. Numerous studies have reported both the total antioxidant and radical scavenging activities of members of the *Allium* genus, ranging from 14.5 to 128 mg AAE/g [22,50] and 11 to 15,000 μg/mL, respectively [33,51].

LE decreased the degranulation of mast cells through both an IgE-independent and -dependent pathway. The inhibitory effect of plant extracts on mast cell degranulation has been reported before. Yoo et al. demonstrated that aged black garlic extract inhibited the release of β-hexosaminidase on HumRBL-2H3 cells [52]. Moreover, Lee et al. showed that the Korean herbal medicine KOTMIN13 inhibited the degranulation of bone marrow mast cells derived [53]. Our results demonstrated that methanolic LE inhibits the degranulation of both HumRBL-2H3 and LAD2 cells in response to immunological and non-immunological stimuli, respectively. Therefore, LE could be useful as a mast cell stabilizer in allergy.

Since respiratory epithelial barrier disruption has been observed in allergic patients, and airborne pollutants are considered as important inductors of lung oxidative stress [54], resulting in altered lung function [55], LE pretreatment protected against the deleterious effect of H_2_O_2_ on TEER values and promoted epithelial barrier maturation. The protective effect of plant extracts against oxidative stress/inflammatory stimuli has been reported for other species, including *Capsicum annuum* (pepper) [56], *Alpinia katsumadai* [57], and *Boswellia serrate* [58].

Exposure to environmental pollutants induces oxidative stress via the activation of transcription factors such as NFĸB and c-Jun [59,60]. Moreover, the intracellular antioxidant defense system includes metabolic enzymes such as GST, which catalyzes the conjugation of the reduced form of glutathione to xenobiotic substrates [61]. We found that LE treatment of epithelial cells prevented the increase in the expression of genes implicated in redox metabolism.

IL-6 is a pro-inflammatory cytokine found at high levels in asthmatic patients [62], and we found significantly lower levels of this cytokine in epithelial cells treated with LE compared to control cells. Previous studies using extracts from other plants of the *Allium* genus have also shown their ability to reduce the production of pro-inflammatory cytokines in vivo. According to Oliveira et al., extracts of onion were able to reduce IL-4 and IL-5 levels in bronchoalveolar lavage of a murine model of asthma [63] and Kim et al. observed that extracts of *A. hookeri* decreased levels of TNF-α in nasal mucosa of a murine model of allergic rhinitis [64].

## 5. Conclusions

In summary, the results obtained in this work suggest that *A. porrum* extract may have potential anti-allergic effects due to its antioxidant and anti-inflammatory properties. More research is needed in order to elucidate the mechanisms and the bioactive compounds responsible for these actions.

## Figures and Tables

**Table 1:** Primers used for RT–PCR amplification of specific genes on Calu-3 cells at ALI.

Gene	Forward Sequence 5′-3′	Reverse Primer 5′-3′
GAPDH	AAAGGGTCATCATCTCTG	GCTGTTGTCATACTTCTC
GST	CGGGCAACTGAAGCCTTTTG	TCAGCGAAGGAGATCTGGTC
c-Jun	GCAAAGAACTTTCCCGGCTG	GGAGAAGCCTAAGACGCAGG
NFκB	TGAGGATGATGAGAATGGAT	CGGAACACAATGGCATAC

GAPDH, glyceraldehyde-3-phosphate dehydrogenase; GST, glutathione S-transferase; c-Jun, Jun proto-oncogene; NFκB, Nuclear factor kappa B subunit.

**Table 2:** Organic acids, carbohydrates, and L-amino acids identified in leek extract (LE) by Gas Chromatography–Mass Spectrometry (GC–MS).

Compound	Retention Time (min)	Molecular Formula	Molecular Mass (g/mol)	R-Match
**Organic acids**				
Boric acid	4.8	H_3_BO_3_	61.8	814
Propanoic acid	5.8	C_3_H_6_O_2_	74.1	899
Acetic acid	6.1	C_2_H_4_O_2_	60.1	849
Phosphoric acid	8.9	H_3_PO_4_	98.0	869
Succinic acid	9.5	C_4_H_6_O_4_	118.09	918
Fumaric acid	10.0	C_4_H_4_O_4_	116.1	776
Nonanoic acid	10.2	C_9_H_18_O_2_	158.2	787
Malic acid	11.8	C_4_H_6_O_5_	134.1	859
Arabinonic acid	13.5	C_5_H_10_O_6_	166.1	729
Palmitic acid	17.8	C_16_H_32_O_2_	256.4	705
Stearic acid	19.1	C_18_H_36_O_2_	284.5	702
Oleic acid	19.7	C_18_H_34_O_2_	282.5	808
**Carbohydrates**				
Arabinofuranose	8.6	C_5_H_10_O	150.1	777
Glucofuranoside	15.4	C_6_H_12_O_6_	180.2	772
D-fructose	15.5	C_6_H_12_O_6_	180.2	809
Mannofuranoside	15.6	C_7_H_14_O_6_	194.2	781
Glucose	17.1	C_6_H_12_O_6_	180.2	790
Galactopyranose	17.1	C_6_H_12_O_6_	180.2	825
**Amino acids**				
L-valine	6.3	C_5_H_11_NO_2_	117.2	798
L-proline	12.2	C_5_H_9_NO_2_	115.1	850

**Table 3:** Total phenol content, antioxidant activity, and 2,2-diphenyl-1-picrylhydrazyl radical scavenging activity IC50 of LE.

	Mean ± SD
**Total phenol content**	2.3 ± 0.2 ^a^
**Total antioxidant activity**	60.2 ± 3.1 ^b^
**DPPH scavenging activity, IC_50_**	289.6 ± 6.1 ^c^

SD, standard deviation. ^(**a**)^ mg GAE/g dry extract; ^(**b**)^ mg AAE/g dry extract; ^(**c**)^ μg/mL.

**Table 4:** Mass spectral data for identification of phenolic compounds in LE by Liquid Chromatography–Mass Spectrometry (LC–IT/MS).

Polyphenol Subclass (Flavonoids)	Compound Name	Retention Time (min)		Positive Ions (*m*/*z*)			
			MW	[M+H]^+^	[M+Na]^+^	[M+K]^+^	In-Source Fragments
flavanols	(-)-Epicatechin ^a^	10.0	290	**291**	--	--	--
flavanols	(+)-Catechin ^a^	12.3	290	**291**	--	--	--
flavonols	Kaempferol derivative ^b^	19.4	742	743	**765**	--	-- [Aglycone+H]^+^ = 287, [Aglycone+H-H_2_O]^+^ = 269
flavonols	Kaempferol derivative ^c^	19.8	902	903	925	--	[(M-Rham)+H]^+^ = **757** [(M-Rham-Glc)+H]^+^ = 595 [(M-Rham-2Glc)+H]^+^ = 433 [Aglycone+H]^+^ = 287, [Aglycone+H-H_2_O]^+^ = 269
flavonols	Quercetin derivative ^b^	18.8	625	627	--	--	[(M-Glc)+H]^+^ = **463** [Aglycone+H]^+^ = 303, [Aglycone+H-H_2_O]^+^ = 285
flavonols	Quercetin derivative ^b^	20.5	609	611	633	**649**	[Aglycone+H]^+^ = 303, [Aglycone+H-H_2_O]^+^ = 285

*m/z* values for the base peak are given in bold type. ^(^**^a^**^)^ Identification by comparison with the pure standard. ^(**b**)^ Identification by in source-fragmentation and by searching in online databases such as FOODB (http://foodb.ca) and METLIN (http://metlin.scripps.edu). ^(^**^c^**^)^ Identification by comparison with bibliography: kaempherol-3-*O*-[rhamnosyl-glucosylglucoside]-7-*O*-rhamnoside [32].
